# Creating a Meaningful Life: Psychobiographical Investigations

**DOI:** 10.5964/ejop.6653

**Published:** 2021-08-31

**Authors:** Claude-Hélène Mayer, Paul J. P. Fouché, Roelf van Niekerk

**Affiliations:** 1 Department of Industrial Psychology and People Management, University of Johannesburg, Johannesburg, South Africa; 2 Faculty of Cultural Studies, European University Viadrina Frankfurt (Oder), Frankfurt, Germany; 3 Department of Psychology, University of the Free State, Bloemfontein, South Africa; 4 Department of Industrial and Organisational Psychology, Nelson Mandela University, Port Elizabeth, South Africa

**Keywords:** psychobiography, meaning, identity, extraordinary individuals

## Abstract

This article serves as the editorial to the Special Issue of Europe’s Journal of Psychology that focusses on “Creating a meaningful life: Psychobiographical investigations.” The introduction provides a brief overview of the articles that offer original and innovative approaches to the growing research area of psychobiography, meaning and identity from different theoretical, methodological, disciplinary and socio-cultural background.

*It does not have to be pretty.*

*It has to be meaningful.*

— *Duane Hanson,*

*American Sculptor, 1925–1996*

Psychobiographical research focuses on the lives of extraordinary individuals and employs psychological theory to clarify and illuminate historically significant events, processes and contributions (Fouché & van Niekerk, 2010). In essence, psychobiographies are psychological biographies that offer explorative psycho-historical descriptions and interpretations of life history data. It is for this reason that the field of psychobiography is often viewed as a sub-division of psycho-history.

During the past four decades psychobiographical research has developed into a vibrant and popular area of research and gained considerable international interest (Elms, 1988, 1994, 2007; Kasser, 2017; Mayer & Kovary, 2019; McRunyan, 1997; Ponterotto, 2015; Schultz & Lawrence, 2017; Wegner, 2020, in press). The study of individuals is an intriguing and illuminating field in psychology that contributes to the understanding of individual personality development within the socio-cultural and historical context (Mayer, Fouché, & van Niekerk, 2021; McAdams, 1994; Ponterotto & Moncayo, 2018). Some psychobiographies also investigate multiple personalities with a common dimension amongst them. An example is the work by Carolina Saccaggi (2015), titled “*Leading the latter-day Saints: Psychobiographical studies of Mormon Prophets.*”

Extraordinary individuals (sometimes also referred to as significant individuals) have been studied from multiple perspectives (Fouché, 2015; Fouché, Nortjé, Welman, & Van Niekerk, 2018; Fouché & van Niekerk, 2010; Mayer & Kovary, 2019; Mayer et al., 2021; Ponterotto, 2017; Ponterotto, Reynolds, Morel, & Cheung, 2015). A range of methodological and theoretical approaches have been used to provide alternative interpretations of entire lives or selected life events (Mayer, 2017; McAdams, 1999; McRunyan, 1997). The meaning of life and how it is defined and created elucidate how actions and experiences are constructed and ultimately, how lives are lived. Psychobiographies on meaning-creation have, however, hardly been published to date (Mayer, 2021; Mayer & Kelley, 2021). In the past psychobiographical studies have often focused on psychoanalytical interpretations of significant individuals’ and their possible psychopathology. On the other end of the continuum, some historical psychobiographies also tended to use religious explanations of “saintly” persons. This is commonly referred to as hagiographies, whereby undue reverence is given to certain individuals. It appears that there has been a move away from both “pathologising” individuals on the one hand and showing undue and undeserved reverence of individuals on the other hand. The focus has shifted towards valuing their psychological strengths and the meaning they create from past life trauma, their creativity and productivity in their various areas of expertise. This entails a more eugraphic approach to undertaking psychobiography.

## Aim of This Special Issue

This special issue aims to fill the gap on meaning-creation in psychobiographical research. It particularly focuses on the creation of meaning to life and significant events in the lives of extraordinary individuals. The aim is to uncover and reconstruct the characteristics, virtues, events, existential forces, life-themes, narratives and talents that enable significant individuals to create meaning in their lives.

## Contributions in This Special Issue

This special issue includes nine psychobiographical investigations on creating a meaningful life from different disciplinary, socio-cultural, historical, political, and economic perspectives. The investigations address and develop previous psychobiographical research by promoting a focus on the question on how to create a meaningful life. The Special Issue further includes a Book Review and this Editorial. The contributions in this Special Issue are written by twenty authors from four continents and seven countries, namely South Africa, Australia, the US, Hungary, Poland, Greece and Germany. It thereby contributes to making psychobiographies an impactful global research topic.

This section provides a brief overview of the articles published in this Special Issue.

This Special issue starts with the article “*The meaning of life and death across Frankl’s life: Archetypal and terror management perspectives*” written by ***Claude-Hélène Mayer, Nataliya Krasovska,*** and ***Paul Fouché.*** It aims to uncover the meaning of life and death across the lifespan of the extraordinary person, Viktor Frankl (1905–1997). The study describes the meaning of life and death through two theoretical approaches: the archetypal analysis based on Carl. Jung’s and Carol Pearson’s work as well as a terror management approach based on the melancholic existentialist work of Ernest Becker. The article evaluates how archetypes and death anxiety interacts and how they built meaning in different stages of Frankl’s lifespan. The theories are discussed and illustrated in the light of Viktor Frankl’s life.

In the article written by ***Hanan Bushkin, Roelf van Niekerk*,** and ***Louise Stroud*** titled “*Searching for meaning in chaos—Viktor Frankl's story*”, the focus is on the search for meaning within unpredictable, life-threatening, and chaotic contexts viewed through the lens of Frankl‘s concept of noö-dynamics. Although Frankl formulated his existential approach to psychological practice before WWII, his experiences in the concentration camps confirmed the view that it is through a search for meaning and purpose in life that individuals can endure hardship and suffering. In a sense, Frankl’s theory was tested in a dramatic way by the tragedies of his life. This article focuses specifically on the period between 1942 and 1945 and explores Frankl’s strategies for attaining meaning. In his theory Frankl proposed that the creation and discovery of meaning can be achieved through creative pursuits, the experience of love, and through an attitudinal value. This investigation indicated that Frankl created meaning by employing at least eight strategies: creative pursuits, serving others, contradicting experiences, committing to decision, establishing spiritual connections, perceiving meaningless tasks through meaning-creation lenses, creating and pursuing goals, and lastly, maintaining an unconditional attitude of strength. The article contributes by shedding light on specific principles and strategies to create meaning in unchangeable circumstances and contexts.

***Carla Nel, Barbara Burnell, Paul, Fouche***, and ***Roelf van Niekerk***, provide in their article “*Meaning and wellness: A comparative psychobiography of the lives of Helen Suzman and Beyers Naudé*” a comparative psychobio­graphical study pregarding the lives of two extraordinary individuals. A comparison of the construction of meaning, as an important aspect of wellness within the holistic wellness model, is made for these South African anti-apartheid activists. Suzman (1917–2009) dedicated her career to opposing apartheid policy as parliamentary politician. Naudé (1915–2004) was a renowned public figure dedicated to the pursuit of social justice in his role as theologian. The holistic wellness model views spirituality as crucial to ascribing meaning to life events and underlines the importance of meaningfulness in enhancing wellness. In addition, the model acknowledges multiple potential sources of meaning. The differences and similarities pertaining to the domains of meaning-making of these two subjects are discussed in this article. The article contributes, through the use of a comparative psychobiographical methodology to the eugraphic exploration of the meaning-making processes of these exemplary individuals.

“*The emperor of fashion’s new starts—Karl Lagerfeld’s creativity in psychobiographical perspective*” is written by ***Claude-Hélène Mayer*** and ***James Kelley***. It deals with the life of the fashion designer Karl Lagerfeld and his creative approach to work and life. The authors apply two specific creativity theories to Lagerfeld’s life and work, namely the mini-c, little-c, Pro-c and Big-C creativity theory and Sternberg’s WICS-model. The article uses a psychobiographical case study design formulated according to a research paradigm of modern hermeneutics. The findings demonstrate the interplay of mini-c, little-c, Pro-c and Big-C creativity throughout the subject’s lifetime, as well as the subject’s application of WICS, both of which led to the subject’s worldwide success.

The article titled “*The episodic man: How a psychobiography of Donald Trump casts new light on research into narrative identity,*” written by ***Dan McAdams***, emphasizes that people create meaning through life narrative. The author presents the life and personality narration of Trump throughout his life time and concludes that Trump has an internalized and evolving story of the self that reconstructs the personal past and imagines the future. Trump lived in the emotionally vivid moment (episode), fighting to win each moment. Seeing him through the lens of the episodic man helps to explain many puzzling features of Donald Trump’s personality, from his charismatic effect on millions of Americans to his penchant for lying and malice to his very strange brand of narcissism. This article gives insight into an in-depth analysis of Trump’s episodic nature, suggesting that people vary not only with respect to the kinds of stories they create for their lives but also with respect to the extent to which they construe life in narrative terms.

In their article titled “*The work of a revolutionary: A psychobiography and careerography of Angela Davis*” authored by ***Jason Reynolds (Taewon Choi), Bridget Anton, Chiroshri Bhattacharjee,*** and ***Megan Ingraham*** reconstruct Davis as a revolutionist, political activist, academic, and writer who has navigated and discussed issues of race, class, gender, and social policies throughout her life. Davis’s story provides a unique understanding of how contemporary issues relate to personal goals and meaning-making in the creation of a career and commitment to a legacy of education and social change. This psychobiography extends beyond the usual to understand more deeply the why of a specific person’s behaviors. It explores Davis’s personal, professional, and representational life using tow theoretical stances, namely the Social Cognitive Career Theory and Simon and Klanderman’s social psychological model for Politicized Collective Identity (PCI). This article thereby expands previously used theoretical models in psychobiography and stimulates new ideas for psychobiography and careerography.

“*The spiritual wellness of an intellectual, novelist, journalist and politician: The meaningful life of Sol Plaatje*” was written by ***Crystal Welman, Paul Fouché, Pravani Naidoo*** and ***Roelf van Niekerk***. The study illustrates Sol Plaatje’s (1876–1932) spiritual wellness across his lifespan. As a South African intellectual, novelist, journalist, and politician, Plaatje was also a founder member of the South African Native National Congress, which later became the African National Congress (ANC). His meaningful life history reflected a significant degree of spiritual wellness. The Wheel of Wellness model (WoW) by Sweeney and Witmer was applied to interpret the biographical evidence of spirituality and meaning in his life. Key findings indicate that spirituality characterized Plaatje’s childhood years and continued to play a pervasive role throughout his adult years. His sense of meaning and purpose was personified in the promotion and preservation of human rights and dignity, that embraced inter-racial love, compassion, and service to others.

In their article titled “*An existential psychobiography of Maya Angelou*”, the authors ***Nadene Harisunker*** and ***Carol du Plessis*** focus on the life of acclaimed author Maya Angelou (1928–2014) through the lens of Frankl’s existential psychology with a specific focus on concepts such as will to meaning and finding meaning in suffering. Angelou’s life autobiographies, poetry, and letters were analysed to contribute to this psychobiography. The inherent search for meaning within Maya Angelou’s life was clearly apparent in the analysis. This study contributes to the development of the research method of psychobiography, with a specific focus on meaning making, using an existential theory for meaning making in Maya Angelou’s life.

The aim of the article titled “*Meaning-making narratives within a puzzle of parts: A psychobiography of Sylvia Plath*”, written by ***Angela Panelatti, Joseph Ponterotto*** and ***Paul Fouché*** is to unveil Sylvia Plath’s (1932–1963) meaning-making narratives, within her life’s puzzle of parts, by utilizing the Internal Family System (IFS) model of Schwartz. The authors use Alexander’s psychobiographical indicators of salience to prioritise and extract relevant evidence from Path’s life. Additionally, a conceptual framework facilitated the longitudinal exploration of Plath’s meaning-making narratives within her life’s puzzle of parts. In light of Plath’s use of her creative genius to address socio-historical issues and injustices against women, her life lends itself to meaning-making narratives to empower and inspire future generations of previously disempowered groups.

The Special Issue closes with a book review on the book of Dan P. McAdams, a well-known psychobiographer from the United States. ***Claude-Hélène Mayer***, an international pioneer in psychobiographical studies and publications, has reviewed the book in her contribution “*The episodic superhero and the purpose of winning—Psychobiographer Dan McAdams on the life and personality of Donald Trump*”. The Book Review provides a comprehensive overview of the psychobiography of Dan McAdams and discusses selected theses provided in the psychobiography on Trump.

## A Future Vision of Psychobiography and Meaningful Lives

This Special Issue presents the latest studies on psychobiography in the context of creating a meaningful life. The studies offer particular insights into specific individuals in socio-cultural contexts from different conceptual and theoretical, as well as methodological stances within psycho-history.

It is suggested that future studies in psychobiography and about meaningful lives can contribute to creating a world and realities of readers who might be inspired by the creation of meaningfulness of extraordinary individuals. It is further assumed, that for a future healthy, balanced world, a new focus on meaningfulness in life is urgently needed. This Special Issue contributes to providing insights into new and original ideas of extraordinary individuals to construct a meaningful life and world from various disciplinary, social and cultural perspectives. The editors therefore hope to contribute stimulating discourses which add value to the readership of EJOP.
